# A systematic study on the treatment of hepatitis B-related hepatocellular carcinoma with drugs based on bioinformatics and key target reverse network pharmacology and experimental verification

**DOI:** 10.1186/s13027-023-00520-z

**Published:** 2023-07-01

**Authors:** Shenghao Li, Liyuan Hao, Xiaoyu Hu, Luya Li

**Affiliations:** 1 Chengdu University of Traditional Chinese Medicine, No. 37 Shi-er-qiao Road, Chengdu, 610075 Sichuan People’s Republic of China; 2 Hospital of Chengdu University of Traditional Chinese Medicine, No. 39 Shi-er-qiao Road, Chengdu, 610072 Sichuan People’s Republic of China; 3 Department of Pharmacy Department, The Fourth Hospital of Hebei Medical University, NO.12, Jian Kang Road, Shijiazhuang, 050010 Hebei People’s Republic of China

**Keywords:** Hepatitis B virus-associated hepatocellular carcinoma, CDK1, CCNB1, Traditional Chinese medicine, Quercetin, Celastrol, Cantharidin

## Abstract

**Background:**

Chronic hepatitis B virus (HBV) infection is the major etiology of hepatocellular carcinoma (HCC). However, the mechanism of hepatitis B-related hepatocellular carcinoma (HBV-related HCC) is still unclear. Therefore, understanding the pathogenesis and searching for drugs to treat HBV-related HCC was an effective strategy to treat this disease.

**Purpose:**

Bioinformatics was used to predict the potential targets of HBV-related HCC. The reverse network pharmacology of key targets was used to analyze the clinical drugs, traditional Chinese medicine (TCM) and small molecules of TCM in the treatment of HBV-related HCC.

**Methods:**

In this study, three microarray datasets totally containing 330 tumoral samples and 297 normal samples were selected from the GEO database. These microarray datasets were used to screen DEGs. And the expression profile and survival of 6 key genes were analyzed. In addition, Comparative Toxicogenomics Database and Coremine Medical database were used to enrich clinical drugs and TCM of HBV-related HCC by the 6 key targets. Then the obtained TCM were classified based on the *Chinese Pharmacopoeia*. Among these top 6 key genes, CDK1 and CCNB1 had the most connection nodes and the highest degree and were the most significantly expressed. In general, CDK1 and CCNB1 tend to form a complex, which is conducive to cell mitosis. Hence, this study mainly studied CDK1 and CCNB1. HERB database was used to predict small molecules TCM. The inhibition effect of quercetin, celastrol and cantharidin on HepG2.2.15 cells and Hep3B cells was verified by CCK8 experiment. The effects of quercetin, celastrol and cantharidin on CDK1 and CCNB1 of HepG2.2.15 cells and Hep3B cells were determined by Western Blot.

**Results:**

In short, 272 DEGs (53 upregulated and 219 downregulated) were identified. Among these DEGs, 6 key genes with high degree were identified, which were AURKA, BIRC5, CCNB1, CDK1, CDKN3 and TYMS. Kaplan–Meier plotter analysis showed that higher expression levels of AURKA, BIRC5, CCNB1, CDK1, CDKN3 and TYMS were associated with poor OS. According to the first 6 key targets, a variety of drugs and TCM were identified. These results showed that clinical drugs included targeted drugs, such as sorafenib, palbociclib and Dasatinib. and chemotherapy drugs, such as cisplatin and doxorubicin. TCM, such as the TCM flavor was mainly warm and bitter, and the main meridians were liver and lung. Small molecules of TCM included flavonoids, terpenoids, alkaloids and glycosides, such as quercetin, celastrol, cantharidin, hesperidin, silymarin, casticin, berberine and ursolic acid, which have great potential in anti-HBV-related HCC. For molecular docking of chemical components, the molecules with higher scores were flavonoids, alkaloids, etc. Three representative types of TCM small molecules were verified respectively, and it was found that quercetin, celastrol and cantharidin inhibited the proliferation of HepG2.2.15 cells and Hep3B cells along concentration gradient. Quercetin, celastrol and cantharidin decreased CDK1 expression in HepG2.2.15 and Hep3B cells, but for CCNB1, only cantharidin decreased CCNB1 expression in the two strains of cells.

**Conclusion:**

In conclusion, AURKA, BIRC5, CCNB1, CDK1, CDKN3 and TYMS could be potential targets for the diagnosis and prognosis of HBV-related HCC. Clinical drugs include chemotherapeutic and targeted drug, traditional Chinese medicine is mainly bitter and warm TCM. Small molecular of TCM including flavonoids, terpenoids and glycosides and alkaloids, which have great potential in anti-HBV-related HCC. This study provides potential therapeutic targets and novel strategies for the treatment of HBV-related HCC.

## Introduction

Hepatocellular carcinoma (HCC) was the third leading cause of cancer-associated mortality worldwide [1]. Major risk factors for HCC were chronic hepatitis B virus infection, alcoholic liver disease and cirrhosis. The main cause of liver cancer was persistent hepatitis B virus (HBV) infection, which had accounted for more than half of liver cancer cases [2–4]. Epigenetic regulation of HBV protein to tumor suppressor genes was involved in the development and progression of hepatitis B-related hepatocellular carcinoma (HBV-related HCC) [5]. However, the mechanism of HBV-related HCC is still unclear. Therefore, in order to improve the survival rate of patients, it is urgent to screen out biomarkers for the diagnosis, prognosis and treatment of HBV-related HCC.

Microarrays have been widely used for molecular diagnosis and for the discovery of new cancer biomarkers, and these techniques were excellent choices for analyzing large gene expression datasets in order to understand the pathogenesis of HBV-related HCC. Many cancer-related databases, such as Gene Expression Synthesis (GEO) and gene expression profile Interaction Analysis (GEPIA), have been developed to analyze the expression of tumor-related genes. Moreover, Kaplan–Meier plotter has been used to analyze gene-related prognoses, contributing to the diagnosis and treatment of cancer.

In addition, the Comparative Toxicogenomics Database (CTD) [6] is an online database that provides information on the interactions between gene products and chemotherapy drugs and diseases. 6 key genes screened from three datasets in GEO database were used for reverse network pharmacology research. CTD, Coremine Medical database and HERB database were used to identified drugs, traditional Chinese medicine (TCM) and small molecules of TCM, respectively. Then, reverse network pharmacology used a variety of public databases for initial disease target selection. Based on the selected targets, effective drugs for a certain disease were explored [7, 8].

Clinical targeted drugs include sorafenib, palbociclib and Dasatinib. And chemotherapy drugs, such as DNA-targeting cisplatin and doxorubicin, are widely used to treat a variety of cancers, including HCC [9]. These clinical drugs benefit many patients. In China, TCM played an important role in treating diseases. TCM had the characteristics of multi-component and multi-target in the treatment of HCC. Therefore, exploring TCM as an effective and low-toxicity anti-cancer drug provided a broad prospect for the prevention and treatment of HCC [10]. In addition, many natural products, such as flavonoids, terpenoids and glycosides and alkaloids, are being evaluated clinically for liver cancer. These compounds inhibit the formation of liver cancer by affecting anti-inflammatory, antioxidant, anti-angiogenesis and anti-metastasis activities [11, 12]. This specific method of finding therapeutic drugs through targets enabled us to find drugs closely related to diseases, which was conducive to faster large-scale drug screening. The aim of this study was to screen out the potential targets of HBV-related HCC, as well as TCM and chemical components for its treatment by integrated analysis, so as to provide scientific reference for revealing the mechanism of HBV-related HCC and a reliable theoretical basis for the of anti-HBV-related HCC development.

## Materials and methods

### Microarray data

These three profiles GSE47197 [13], GSE84402 [14] and GSE14520 [15] were obtained from the Gene Expression Omnibus (GEO, http://www.ncbi.nlm.nih.gov/geo/). Data from GSE47197 was based on GPL16699 platform and included 61 HBV-related HCC tissues and 63 normal tissues. Data from GSE84402 was based on GPL4133 platform and included 14 HBV-related HCC tissues and 14 normal tissues. Data from GSE14520 was based on GPL3921 platform and included 225 HBV-related HCC tissues and 220 normal tissues.

### Screening for DEGs

The identification of differentially expressed genes (DEGs) between HBV-related HCC and normal tissues was performed in GEO2R (https://www.ncbi.nlm.nih.gov/geo/geo2r/) [16]. As a cutoff criterion, |log FC|> 1.0 and adjusted *P*‑value (adjust‑*P*) < 0.05 were used to identified genes and visualized by volcano plots (http://www.bioinformatics.com.cn/). These three profiles were visualized using the Venn diagram webtool (bioinformatics. psb.ugent.be/webtools/Venn/).

### PPI network and key genes analysis

Search Tool for the Retrieval of Interacting Genes/Proteins (STRING version 11.5) (https://string-db.org/) [17] online database was used to constructed protein-protein interaction (PPI) network. Then, the PPI network was visualized by Cytoscape version 3.8.2 software (https://cytoscape.org/). In addition, the key subnetwork and degree of each protein node was evaluated by MCODE and Cytohubba, respectively. The top 6 genes (AURKA, BIRC5, CCNB1, CDK1, CDKN3 and TYMS) were identified as the key genes.

### Verification of the key genes in GEPIA and UALCAN database

Gene Expression Profiling Interactive Analysis (GEPIA) (http://gepia.cancer-pku.cn/) [18], a cancer big data analysis website, was used to analyze differential expression from The Cancer Genome Atlas (TCGA) and the Genotype-Tissue Expression (GTEx) portal. The cut-off criteria of |log2FC|= 1 and *P*-value ≤ 0.01 were considered statistically significant. UALCAN(http://ualcan.path.uab.edu) [19], a user friendly interactive network resources, was used to analyze cancer transcriptome data (TCGA and MET500 transcriptome sequencing). Therefore, we analyzed the different stages of 6 key genes.

### Protein expressions of key genes in the HPA database

The Human Protein Atlas (HPA) version 21.0 website (https://www.proteinatlas.org/) [20] provided researchers with a wealth of proteomic data on human tissues and cells. HPA database was used to validate the immunohistochemistry of 5 key genes in HCC tissues and normal tissues. However, image of CDKN3 was not found.

### Survival analysis of key genes

Kaplan-Meier plotter (https://kmplot.com/analysis/) [21] was performed overall survival (OS) analysis of 6 key genes. Kaplan-Meier plotter (https://kmplot.com/analysis/) predicted survival time, providing a comparison of survival rates between patients with low and high expression levels of a particular gene, and calculating log‑rank *P*‑value and hazard ratios (HR) with 95% confidence intervals. We performed the survival curves of 6 key genes at 48 months to prevent crossover.

### Drug-gene interaction network analysis

The Comparative Toxico genomics Database [6] (CTD, http://ctdbase.org/) is an online database that provides information on the interactions between gene products and chemotherapy drugs and diseases. It was used to construct chemotherapeutic drug-gene interaction networks [22]. And was visualized using Cytoscape software 3.8.2.

### Analysis of corresponding chemical components of key targets

HERB (http://herb.ac.cn/) database [23] gathered and reanalyzed 1,037 high throughput sequencing experiments that were treated by herbs/ingredients, which was used to analyze the components corresponding to key targets, including the components verified by literature and predicted by multiple databases.

### Key target prediction of traditional Chinese medicine and its classification and characteristics

Coremine Medical database [ 24] (http://www.coremine.com/medical/?locale=zh_CN#search?ids=518629&tt=7679&org=hs&i=518629) was a medical information retrieval platform. Top 6 genes related to HBV-related HCC were input into Coremine Medical database for TCM screening. Then the obtained TCM were imported into TCMSP database. Finally, the mainly TCM were obtained. The regularity of TCM treatment of HBV-related HCC was obtained by frequency analysis. Various properties such as channel tropism, flavor, and property were investigated and were visualized using bioinformatics tools (http://www.bioinformatics.com.cn/).

### Molecular docking

The structure of small molecule was downloaded from Pubchem (https://pubchem.ncbi.nlm.nih.gov/) database, and the structure of AURKA, BIRC5, CCNB1, CDK1, CDKN3 and TYMS were downloaded from PDB (https://www.rcsb.org/) database. Later, the target proteins were imported into Auto Dock Tools 1.5.6 for hydrogenation, charge calculation, and non-polar hydrogen combination, and then the result was stored in PDBQT format.

### Cell line and drug treatment

HepG2.2.15 cells and Hep3B2.1-7 (Hep3B) cells were purchased from iCell Bioscience Inc. These cells were cultured in Cell specific culture-medium (icell-h091-001b) at 37 ℃ and 5% CO_2_ in an atmosphere of 100% humidity. Quercetin (Lot. # 131,328) and Celastrol (Lot. # 145,154) were purchased from TargetMol Chemicals Inc. Cantharidin (Lot. # GN10058) was purchased from GlpBio Technology. HepG2.2.15 and Hep3B cells were treated with quercetin, celastrol and cantharidin for 24 h or 48 h, respectively.

### Cell viability assay

HepG2.2.15 cells and Hep3B cells were seeded in 96-well plates (5 × 10^4^ cells/well). HepG2.2.15 cells were treated with quercetin (100 μM, 200 μM, 300 μM, 400 μM), celastrol (0.1 μM, 0.5 μM, 1 μM, 2 μM), cantharidin (3 μM, 6 μM, 9 μM, 12 μM) at different concentration for 24 h, and 48 h, respectively. Hep3B cells were treated with quercetin (100 μM, 200 μM, 300 μM, 400 μM), celastrol (0.1 μM, 0.5 μM, 1 μM, 2 μM), cantharidin (1 μM, 2 μM, 3 μM, 4 μM) at different concentration for 24 h, and 48 h, respectively. Cell viability was measured using Cell Counting Kit-8 (CCK-8, Cat. # BS350B, Biosharp) according to manufacturer's protocol. Finally, optical density (OD) was monitored at 450 nm. IC50 values were obtained from the cytotoxicity curves using the ELx800 software.

### Western blot analysis

HepG2.2.15 cells and Hep3B cells were seeded in 6-well plates (1 × 10^5^ cells/well) and treated as described above. In brief, they were washed in PBS, and the cells were directly lysed in a SDS sample buffer. Protein concentration was determined by a BCA method (P0013, Beyotime Biotechnology.). The primary antibodies were rabbit anti-CDK1 polyclonal antibody (Cat. # CY5061, Abways, diluted to 1:2000), anti-CCNB1 polyclonal antibody (Cat. # CY5378, Abways, diluted to 1:2000), and β-actin antibody (Cat. # AC026, abclonal, diluted to 1:50,000). The secondary antibodies were goat anti-rabbit (S0001, affbiotech, diluted by 1:5000). The results were analyzed using SPSS 17.0 software (SPSS, Chicago, IL).

### Statistical analysis

Using GEO2R, the adjusted *P* values of genes from the GEO were calculated. Genes with an adjusted *P* value < 0.05 was regarded as statistically significant. For the Kaplan‐Meier plotter, TIMER and GEPIA analysis, hazard ratios (HR) with 95% confidence intervals and log-rank *P* values were calculated. *P* value < 0.05 was taken as a statistically significant difference.

## Results

### Identification of DEGs and PPI network construction

**Fig. 1:**
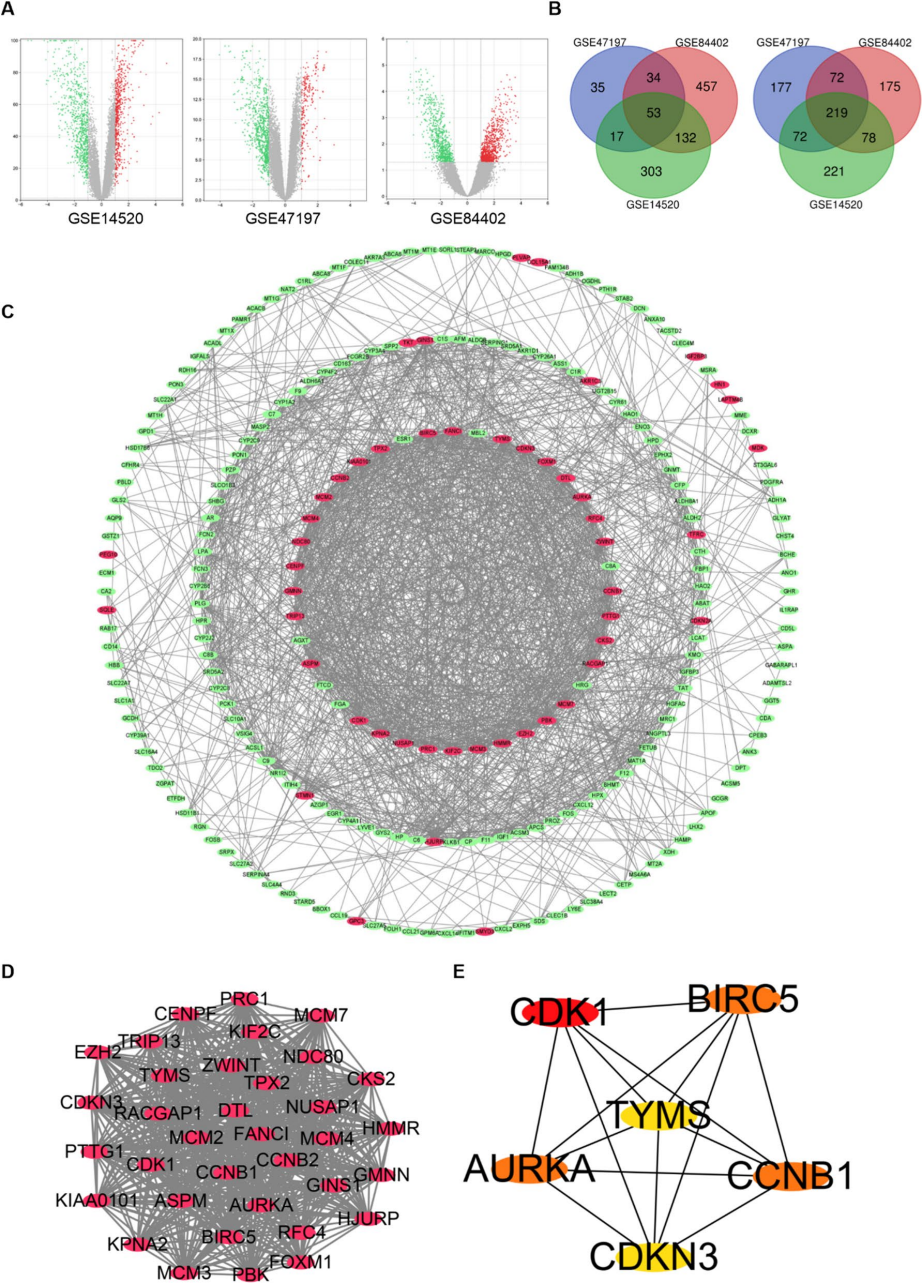
**Volcano plots and Venn diagrams of DEGs selected from three datasets.** A. Volcano plots of DEGs from GSE47197, GSE84402 and GSE14520 datasets. The red dots and green dots were up-regulated and down-regulated DEGs. B. Venn diagram of upregulated (left) and downregulated (right) DEGs based on the three datasets, including 53 upregulated genes and 219 downregulated genes. C. The PPI network was constructed using STRING and visualized by Cytoscape. The red and green circulars represented up and down, respectively. D. The key modules were identified using the MCODE plugin of Cytoscape. E. The 6 key genes were screened in the PPI network by Cytohubba plugin of Cytoscape based on degree. These key genes were displayed from red (high degree value) to yellow (low degree value)

Gene expression profiles of HBV-related HCC and normal liver tissues were obtained from GSE47197, GSE84402 and GSE14520. The results showed that 679, 1220 and 1095 DEGs were identified in GSE47197, GSE84402 and GSE14520, respectively. And the data of each gene expression profile were visualized by volcano plot (Fig. 1A). Subsequently, 53 overlapping upregulated genes and 219 overlapping downregulated genes were screened using Venn online (Fig. 1B). The PPI network was performed via the STRING database and was visualized Cytoscape software, including 247 nodes and 1652 edges. Green and red circulars indicated downregulated and upregulated genes, respectively (Fig. 1C). Then, MCODE analysis was used to detect the most important modules of PPI network. The higher the connection value of the node was, the higher the degree of network connection was, and the greater the correlation degree with disease was (Fig. 1D). The 6 key genes with high degree in HBV-related HCC, including AURKA, BIRC5, CCNB1, CDK1, CDKN3 and TYMS were identified (Fig. 1E).

### Abnormal expression of 6 key genes in HBV-related HCC

**Fig. 2:**
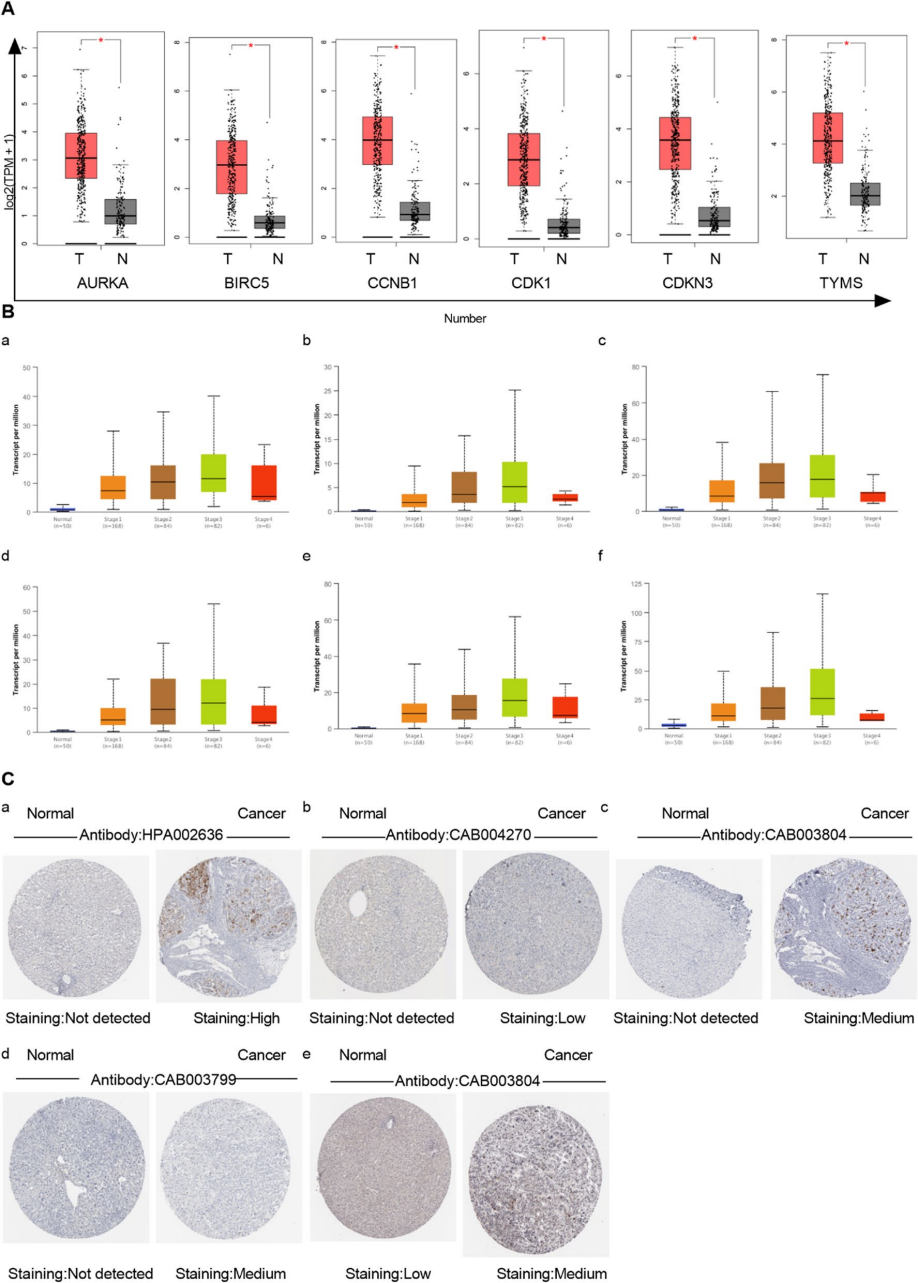
**Abnormal expression of 6 key genes in HBV-related HCC.** A. 6 key genes were more highly expressed in liver cancer tissues compared with those in the normal tissues in GEPIA database. The red and gray boxes represent cancer and normal tissues, respectively. B. The stage of (a) AURKA, (b) BIRC5, (c) CCNB1, (d) CDK1, (e) CDKN3 and (f) TYMS using UALCAN database. C. The immunohistochemical images of (a) AURKA, (b) BIRC5, (c) CCNB1, (d) CDK1, (e) TYMS in liver and HCC tissues using the HPA database

Further, TIMER database was used to analysis. To confirm differential expression at the transcriptional level, the expression levels of 6 key genes were performed using GEPIA database. The results revealed that AURKA, BIRC5, CCNB1, CDK1, CDKN3 and TYMS were significantly upregulated in tumor tissues (Fig. 2A).

The results showed that these 6 genes expression in HBV-related HCC tissues was higher than that in normal tissues, based on different pathological stages (Fig. 2B). Therefore, the expression levels of AURKA, BIRC5, CCNB1, CDK1, CDKN3 and TYMS served as diagnostic markers for liver cancer patients. In addition, overexpression of these 6 key genes was also associated with advanced pathological stage. Therefore, these results suggested that the expression of these 6 key genes played a significant role in the development of HBV-related HCC.

Subsequently, using the HPA database, the protein expression levels of 6 key genes were analyzed. Immunohistochemistry staining of HPA database showed that except CDKN3 was not found, AURKA, BIRC5, CCNB1, CDK1 and TYMS proteins were not or low expressed in normal liver tissues, while low (BIRC5), medium (CCNB1, CDK1 and TYMS) and high expression (AURKA) were shown in HBV-related HCC tissues (Fig. 2C).

### Kaplan–Meier survival analysis of 6 key genes

**Fig. 3:**
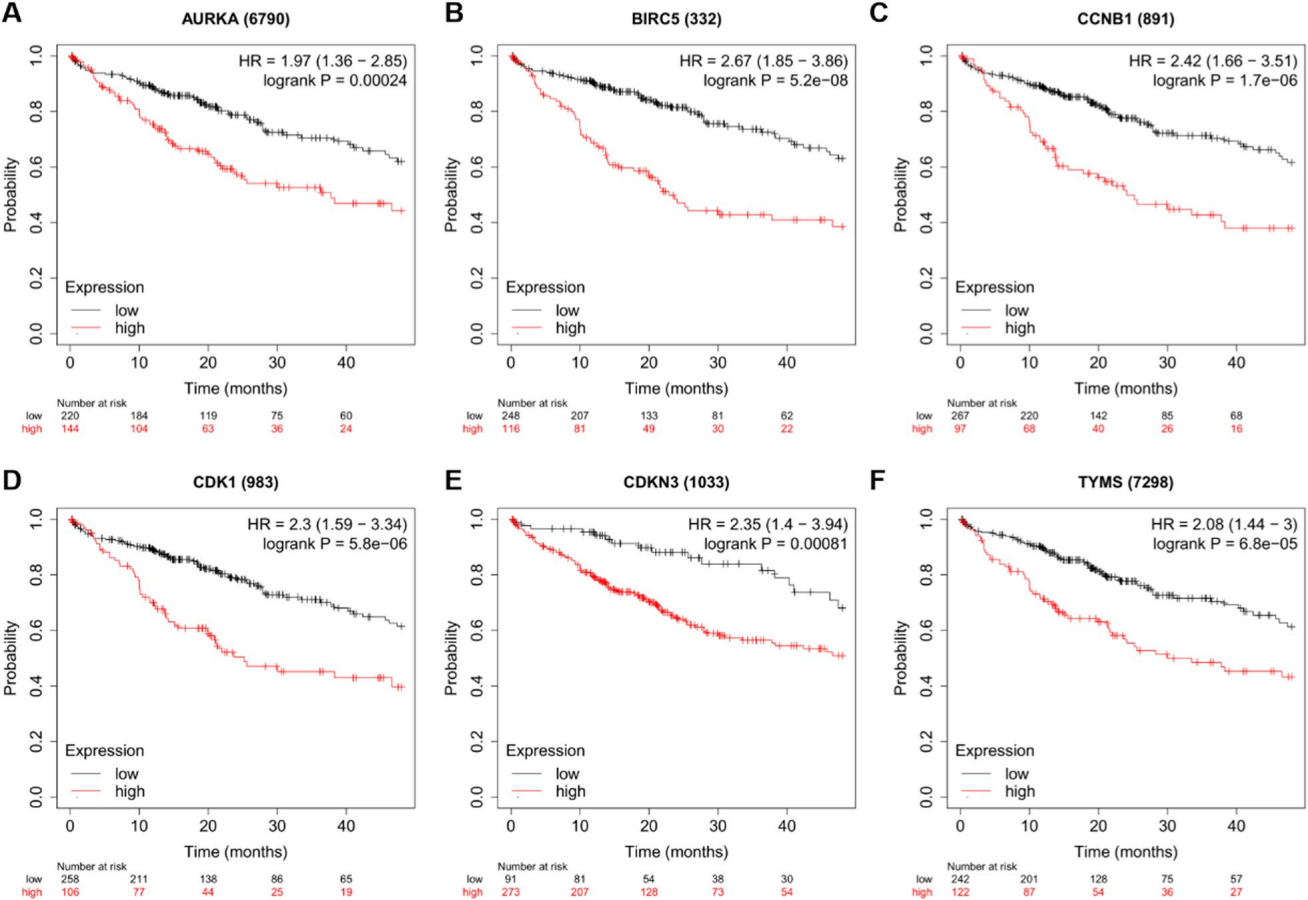
Survival analysis of 6 key genes (A) AURKA, (B) BIRC5, (C) CCNB1, (D) CDK1, (E) CDKN3 and (F) TYMS

To further illustrate whether 6 key genes were potentially prognostic markers for HBV-related HCC, OS was analyzed using Kaplan–Meier plotter (http://kmplot.com/analysis/). AURKA [hazard ratio(HR) = 1.97(1.36-2.85), log-rank *P* = 0.00024], BIRC5 [hazard ratio(HR) = 2.67(1.85-3.86), log-rank *P* = 5.2e-08], CCNB1 [hazard ratio(HR) = 2.42(1.66-3.51), log-rank *P* = 1.7e-06], CDK1 [hazard ratio(HR) = 2.3(1.59-3.34), log-rank *P* = 5.8e-06], CDKN3 [hazard ratio(HR) = 2.35(1.4-3.94), log-rank *P* = 0.00081] and TYMS [hazard ratio(HR) = 2.08(1.44-3), log-rank *P* = 6.8e-05] were highly expressed and associated with poor OS time (Fig. 3). Multiple databases showed that AURKA, BIRC5, CCNB1, CDK1, CDKN3 and TYMS could be prognostic markers for HBV-related HCC.

### Prediction of clinical drug use and key target action by CTD database

**Fig. 4:**
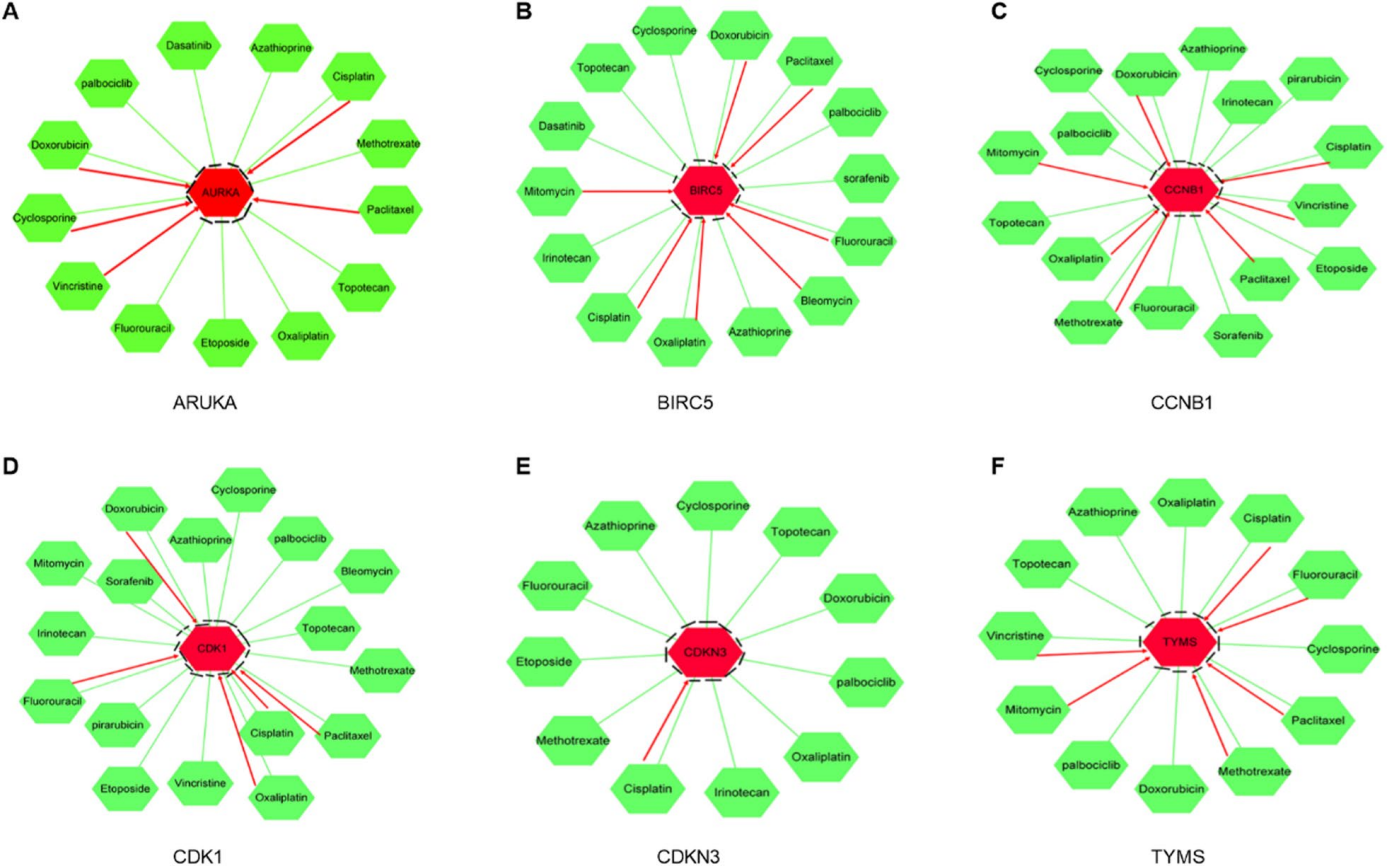
The drug-gene interaction network of chemotherapeutic drugs and 6 key genes, (A) AURKA, (B) BIRC5, (C) CCNB1, (D) CDK1, (E) CDKN3 and (F) TYMS, was constructed using CTD database

Subsequently, several drugs were found to affect HBV-related HCC, a drug-gene PPI network was constructed using CTD database. The result showed that all drugs affected the expression levels of 6 key genes (AURKA, BIRC5, CCNB1, CDK1, CDKN3 and TYMS), but only Topotecan, Palbociclib, and Azathioprine reduced the expression levels of the 6 key genes. Interestingly, cisplatin had both promoting and inhibiting effects on 6 key genes. Targeted drugs include sorafenib, Dasatinib, Palbociclib, etc. Sorafenib inhibited BIRC5, CCNB1 and CDK1; Dasatinib inhibited AURKA and BIRC5; palbociclib inhibited AURKA, BIRC5, CCNB1, CDK1, CDKN3 and TYMS expression (Fig. 4).

### Meridian tropisms, tastes and properties of TCM against HBV-related HCC

**Fig. 5:**
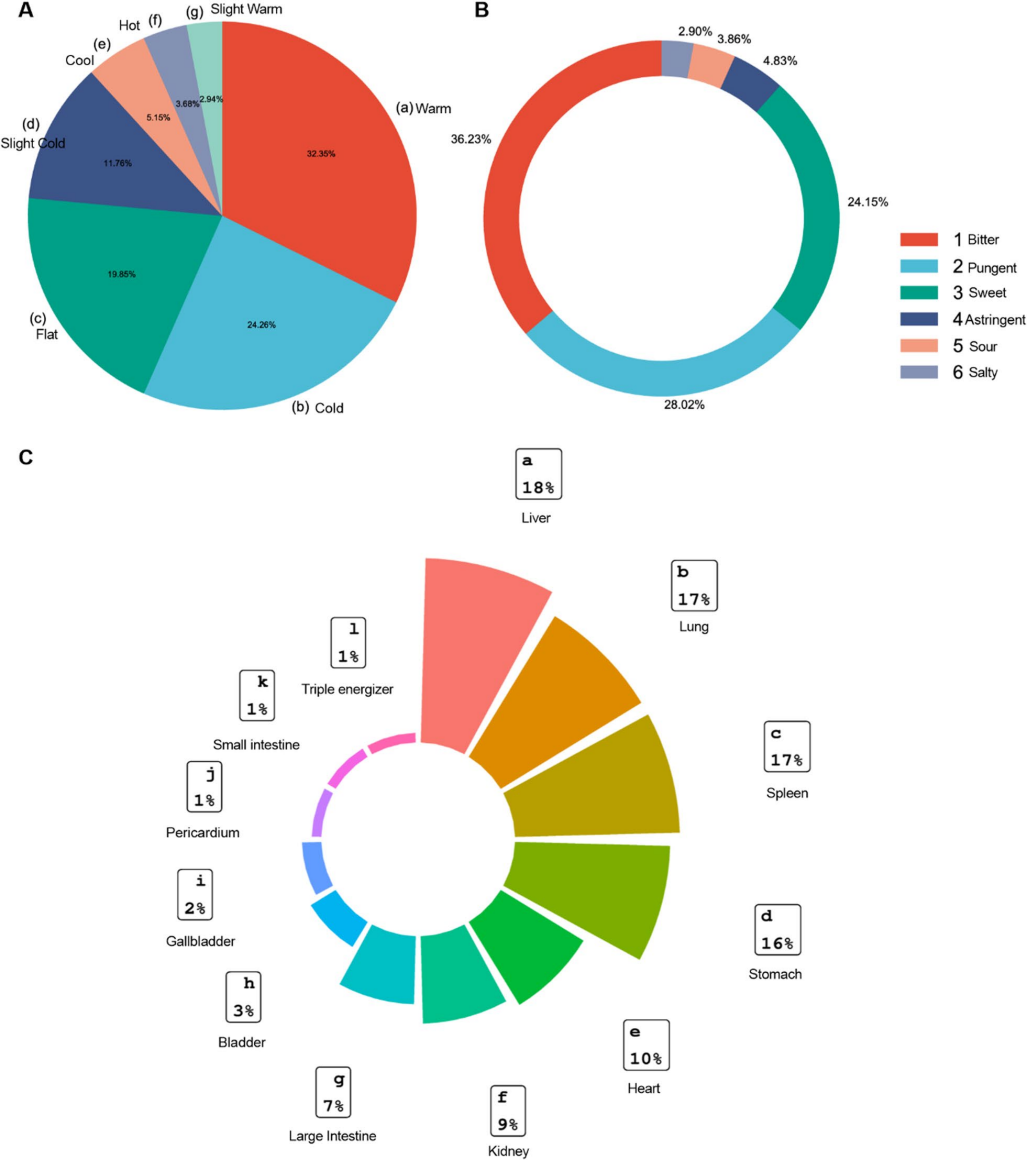
**Properties, tastes, and meridian tropisms of TCM against HBV-related HCC.** A. Properties of herbs against HBV-associated HCC. B. Tastes of herbs against HBV-associated HCC. C. Meridian tropism of herbs against HBV-associated HCC

We not only focus on target screening, but also combined with the corresponding herbs. The result showed that a total of 243 herbs were enriched by reverse enrichment of 6 key targets. To evaluate the reliability of the final results, the TCM recommended in the Guidelines for the Diagnosis and Treatment of primary liver Cancer (2022 edition) and the Clinical Guidelines for the Diagnosis and Treatment of chronic Hepatitis B (2018 edition) were summarized. After removing duplicates, 122 TCM were listed in the two guidelines. After comparison, 39 out of 122 herbs (almost 32%) were included in the 243 herbs, indicating that TCM produced by target-TCM reverse network pharmacology were not out of clinical practice. Among these 243 kinds of TCM, 136 were included in the *Chinese Pharmacopoeia* (2020 edition). Based on the results of frequency analysis, the medication rules of 136 herbs in the *Chinese Pharmacopoeia* (2020 edition) were summarized. In TCM, the nature of herbal medicine referred to its efficacy, divided into cold, cool, even, hot, and warm. The nature of TCM was the basis of its analysis and clinical application. As shown in the Fig. 5A, the main characters of 136 kinds of TCM were warm (32.35%), followed by cold (24.26%), and the total proportion of these two drugs accounted for more than half of all TCM. According to the theory of TCM, warm herbs had the effect of warming and nourishing qi and dispelling evil spirits. Secondly, cold herbs had scavenging and inhibiting effects [25].

Subsequently, we analyzed five flavors: pungent, sweet, sour, bitter and salty. The taste of the herb was mainly bitter, and then pungent (Fig. 5B). Bitter taste had the effect of clearing moisture and reducing fire, while pungent herb had the effect of dispersing outward and upward. Bitter medicine combined with pungent removed blood stasis. This improved blood circulation and relieve pain [25].

The theory of meridian tropism played an important role in clinical of TCM. The frequency of meridian return of TCM could be classified and determined by fan-shaped chart. From the meridional aspect, half of TCM was liver meridian, which was consistent with the disease areas of HBV-related HCC. The second meridian was the lung meridian (Fig. 5C). The liver was responsible for regulation and promotion. The lung was responsible for purification and descent [26]. The two organs complement each other to coordinate qi, blood and body fluid, and restore non-pathological state [27]. Our results also found that therapeutic effects were predominant for the liver, lung, spleen, and kidney, reflecting that TCM was holistic.

### Key target reverse prediction of small molecule of TCM

**Fig. 6:**
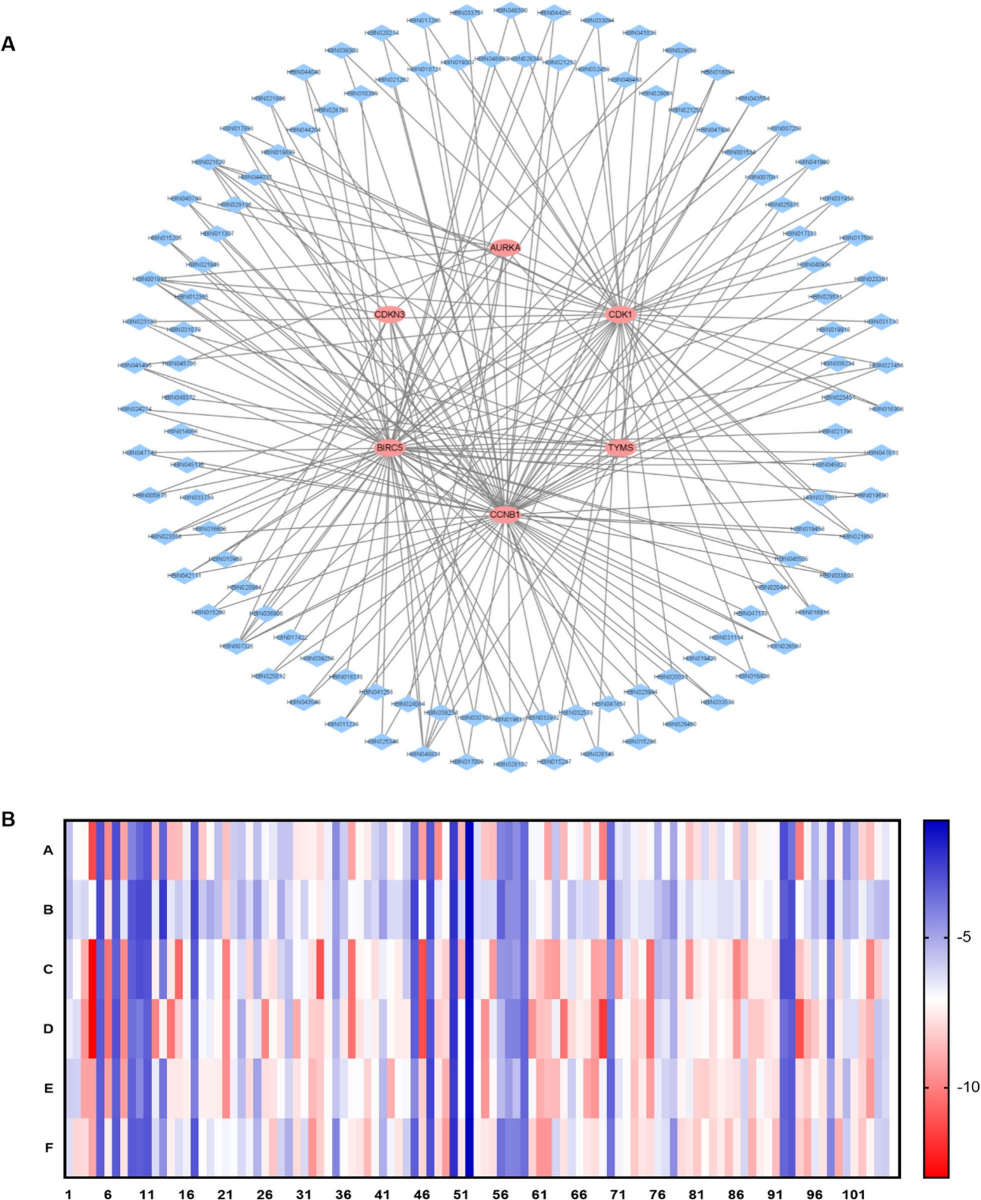
Key target reverse prediction of small molecule of TCM. A Target-component PPI network. Red circles represented target and blue diamonds represented component. B. Heat map of docking results between 105 chemical components (X-axis) and 6 targets (Y-axis). Blue to red indicates the docking score from small to large.

Subsequently, the corresponding chemical components of 6 key targets were analyzed, including the chemical components verified by literature and the chemical components predicted by multiple databases (Table 1). The result indicated that 6 key targets of HBV-related HCC were identified in 115 TCM chemical components. A target-components PPI network was constructed, including 121 nodes and 196 edges (Fig. 6A). Red circles indicated targets and blue diamonds indicated chemical components. The edge indicated the interaction between the target and the chemical components. The larger the node, the greater the connectivity.

**Table 1:** In the Herb database, the components corresponding to key targets

Reference Mining		
Target	Ingredient id	Ingredient name
AURKA	HBIN029196	Hesperidin
AURKA	HBIN045705	Taxol
BIRC5	HBIN039256	Periplocin
BIRC5	HBIN038254	Oridonin
BIRC5	HBIN033972	Lycopene
BIRC5	HBIN023994	Dihydrotanshinone i
BIRC5	HBIN047173	Triptolide
BIRC5	HBIN048300	Withaferin a
BIRC5	HBIN047613	Ursolic acid
BIRC5	HBIN029531	Honokiol
BIRC5	HBIN001534	1,4-naphthoquinone
CCNB1	HBIN021850	Cucurbitacin e
CCNB1	HBIN023361	Deoxyelephantopin
CCNB1	HBIN043554	Secalonic acid d
CCNB1	HBIN031114	Isorhamnetin
CCNB1	HBIN017285	Atractylenolide i
CCNB1	HBIN017893	Berberine
CDK1	HBIN021850	Cucurbitacin e
CDK1	HBIN021850	Cucurbitacin e
CDK1	HBIN040936	Protopine
CDK1	HBIN023361	Deoxyelephantopin
CDK1	HBIN043554	Secalonic acid d
CDK1	HBIN041038	Pseudolaric acid B
CDK1	HBIN048300	Withaferin a
CDK1	HBIN017285	Atractylenolide i
CDK1	HBIN017893	Berberine

Database mining		
AURKA	HBIN001991	17-beta-estradiol
AURKA	HBIN007325	3,4-benzopyrene
AURKA	HBIN015963	Anacardic acid
AURKA	HBIN016816	Aristololactam A IIIa
AURKA	HBIN016908	Arsenicum
AURKA	HBIN021620	Coumestrol
AURKA	HBIN024084	Dimethyl sulfoxide
AURKA	HBIN028102	Glycerin
AURKA	HBIN046831	Trans-resveratrol
BIRC5	HBIN001991	17-beta-estradiol
BIRC5	HBIN005875	2'-methoxy-3,4,4'-trihydroxychalcone
BIRC5	HBIN007208	3,3-thiobis-1-propene
BIRC5	HBIN007325	3,4-benzopyrene
BIRC5	HBIN010399	[4]-gingerol
BIRC5	HBIN012365	[6]-gingerol
BIRC5	HBIN015205	Allitridin
BIRC5	HBIN015247	Allyl disulfide
BIRC5	HBIN015260	Allyl monosulfide
BIRC5	HBIN016606	Archin
BIRC5	HBIN017009	Arundine
BIRC5	HBIN017422	Azadirachtin A
BIRC5	HBIN018373	Betulic acid
BIRC5	HBIN019611	Cantharidin
BIRC5	HBIN019690	Capsaicin
BIRC5	HBIN020031	Celastrol
BIRC5	HBIN020444	Chrysazin
BIRC5	HBIN021620	Coumestrol
BIRC5	HBIN021795	Cryptotanshinone
BIRC5	HBIN021986	Curcumine
BIRC5	HBIN023188	Delta(9)-tetrahydrocannabinol
BIRC5	HBIN023558	Diallyldisulfide
BIRC5	HBIN025346	(-)-epigallocatechin-3-gallate
BIRC5	HBIN026567	Flavopiridol
BIRC5	HBIN027456	Genistein
BIRC5	HBIN028349	Gossypin
BIRC5	HBIN028783	Hanfangchin a
BIRC5	HBIN031079	Isopsoralen
BIRC5	HBIN033094	Licochalcone B
BIRC5	HBIN033578	Lovastatin
BIRC5	HBIN033739	Lupan-3-one
BIRC5	HBIN033803	Luteolin
BIRC5	HBIN036905	Nicotine
BIRC5	HBIN040799	Progesterone
BIRC5	HBIN041253	Puerarin
BIRC5	HBIN041495	Quercetin
BIRC5	HBIN041980	Realgar
BIRC5	HBIN042111	Resveratrol
BIRC5	HBIN044035	Silybin b
BIRC5	HBIN044040	Silymarin
BIRC5	HBIN045505	Tanshinone i
BIRC5	HBIN045822	Tea polyphenols
BIRC5	HBIN046831	Trans-resveratrol
BIRC5	HBIN047613	Ursolic acid
BIRC5	HBIN047746	Vanilloid
BIRC5	HBIN047936	Vinblastine
BIRC5	HBIN048463	Xanthotoxin
BIRC5	HBIN048883	Zerumbone
CCNB1	HBIN001991	17-Beta-estradiol
CCNB1	HBIN006234	2-Octynal
CCNB1	HBIN007081	3,3',4',5,5',7-hexahydroxyflavone
CCNB1	HBIN007208	3,3-thiobis-1-propene
CCNB1	HBIN007325	3,4-benzopyrene
CCNB1	HBIN010721	4-methylsulfinyl butyl isothiocyanate
CCNB1	HBIN011239	5, 7, 4′-trihydroxyflavone
CCNB1	HBIN011307	5,7-dihydroxyflavone
CCNB1	HBIN014956	Ajoene
CCNB1	HBIN015205	Allitridin
CCNB1	HBIN015247	Allyl disulfide
CCNB1	HBIN015260	Allyl monosulfide
CCNB1	HBIN015286	Aloeemodin
CCNB1	HBIN016408	Apigenin
CCNB1	HBIN017009	Arundine
CCNB1	HBIN017508	Baicalein
CCNB1	HBIN017893	Berberine
CCNB1	HBIN018094	Beta-elemene
CCNB1	HBIN019425	Calycosin
CCNB1	HBIN019458	Cambogin
CCNB1	HBIN019690	Capsaicin
CCNB1	HBIN019918	Catechin
CCNB1	HBIN020274	Chelidonine
CCNB1	HBIN021250	Coixan A
CCNB1	HBIN021252	Coixan C
CCNB1	HBIN021262	Colchine
CCNB1	HBIN021620	Coumestrol
CCNB1	HBIN021845	Cucurbitacin b
CCNB1	HBIN021986	Curcumine
CCNB1	HBIN023558	Diallyldisulfide
CCNB1	HBIN024274	Dithioerythritol
CCNB1	HBIN025012	Ellipticine
CCNB1	HBIN026145	Eupatilin
CCNB1	HBIN026460	Ferrum
CCNB1	HBIN026567	Flavopiridol
CCNB1	HBIN027456	Genistein
CCNB1	HBIN031730	Kaemferol
CCNB1	HBIN031958	Kaempferol-7-o-glucoside
CCNB1	HBIN032573	L-alpha-amino-delta-hydroxyvaleric acid
CCNB1	HBIN033578	Lovastatin
CCNB1	HBIN033751	Lupeol
CCNB1	HBIN033803	Luteolin
CCNB1	HBIN038303	Oroxylin a
CCNB1	HBIN040799	Progesterone
CCNB1	HBIN041038	Pseudolaric acid B
CCNB1	HBIN041495	Quercetin
CCNB1	HBIN041980	Realgar
CCNB1	HBIN042111	Resveratrol
CCNB1	HBIN043546	Se
CCNB1	HBIN044035	Silybin b
CCNB1	HBIN044037	Silydianin
CCNB1	HBIN044040	Silymarin
CCNB1	HBIN045135	Sulforathane
CCNB1	HBIN046831	Trans-resveratrol
CCNB1	HBIN047746	Vanilloid
CDK1	HBIN001991	17-beta-estradiol
CDK1	HBIN005875	2'-methoxy-3,4,4'-trihydroxychalcone
CDK1	HBIN007325	3,4-benzopyrene
CDK1	HBIN011239	5, 7, 4′-trihydroxyflavone
CDK1	HBIN015247	Allyl disulfide
CDK1	HBIN015286	Aloeemodin
CDK1	HBIN016408	Apigenin
CDK1	HBIN016816	Aristololactam A IIIa
CDK1	HBIN016908	Arsenicum
CDK1	HBIN017333	Atropin
CDK1	HBIN017508	Baicalein
CDK1	HBIN018094	Beta-elemene
CDK1	HBIN019307	Caffeine
CDK1	HBIN019899	Casticin
CDK1	HBIN020274	Chelidonine
CDK1	HBIN021620	Coumestrol
CDK1	HBIN023188	Delta(9)-tetrahydrocannabinol
CDK1	HBIN023558	Diallyldisulfide
CDK1	HBIN025012	Ellipticine
CDK1	HBIN025346	(-)-epigallocatechin-3-gallate
CDK1	HBIN026145	Eupatilin
CDK1	HBIN026460	Ferrum
CDK1	HBIN026567	Flavopiridol
CDK1	HBIN027003	Galangin
CDK1	HBIN027456	Genistein
CDK1	HBIN031730	Kaemferol
CDK1	HBIN031958	Kaempferol-7-o-glucoside
CDK1	HBIN032459	L20
CDK1	HBIN033094	Licochalcone B
CDK1	HBIN033751	Lupeol
CDK1	HBIN038303	Oroxylin a
CDK1	HBIN040799	progesterone
CDK1	HBIN041495	Quercetin
CDK1	HBIN042111	Resveratrol
CDK1	HBIN043546	Se
CDK1	HBIN046831	Trans-resveratrol
CDKN3	HBIN001991	17-beta-estradiol
CDKN3	HBIN007325	3,4-benzopyrene
CDKN3	HBIN020984	Citric acid
CDKN3	HBIN021620	Coumestrol
CDKN3	HBIN028102	Glycerin
CDKN3	HBIN029658	Hydrargyrum
CDKN3	HBIN030108	Lndirubin
CDKN3	HBIN046831	Trans-resveratrol
CDKN3	HBIN047457	Uccinic acid
TYMS	HBIN001991	17-beta-estradiol
TYMS	HBIN007325	3,4-benzopyrene
TYMS	HBIN021620	Coumestrol
TYMS	HBIN023188	Delta(9)-tetrahydrocannabinol
TYMS	HBIN023401	Deoxyuridine
TYMS	HBIN024274	Dithioerythritol
TYMS	HBIN025875	Ethyl aldehyde
TYMS	HBIN028061	Glutathion
TYMS	HBIN028102	Glycerin
TYMS	HBIN029658	Hydrargyrum
TYMS	HBIN044204	S-methyl mercapto-l-cysteine
TYMS	HBIN046831	Trans-resveratrol
TYMS	HBIN048372	Wogonin

Reference mining means that the information in the following table was gathering from curated references. Database mining means that the information in the following table was collected from multiple databases

Molecular docking could predict binding modes of small molecules of TCM with target proteins and predict molecular interactions. Taking into account the 3D structures not available in the PDB database, docking studies were conducted on 6 targets and 105 components. The average binding energy of 630 receptor-ligand docking results was about − 6.61 kcal/mol, indicating that these 105 chemical components had good binding affinity with 6 targets (Fig. 6B) (Table 2). The docking energy scores of 59 kinds of small molecules of TCM with 6 targets were greater than − 6.0 kcal/mol, mainly including flavonoids, glycosides and alkaloids and terpenoids, etc. Representative components of the three categories include quercetin, celastrol and cantharidin. Three kinds of small molecules were verified by experiments.

**Table 2:** Molecular docking binding energy results (kcal/mol)

Number	Ligands/Receptors	AURKA (5dpv)	BIRC5 (3ueh)	CCNB1(4y72)	CDK1(5hq0)	CDKN3(1fq1)	TYMS (4e 28)
1	2-octynal	− 5.6	− 5.3	− 6	− 6.6	− 5.9	− 6.3
2	17-beta-estradiol	− 7.1	− 6.4	− 7.1	− 6.3	− 6	− 8
3	3,3-thiobis-1-propene	− 7.2	− 6.1	− 8.9	− 9.6	− 9.2	− 7.9
4	3,4-benzopyrene	− 11.3	− 7.1	− 13	− 13	− 9.2	− 9.1
5	5,7-dihydroxyflavone	− 3	− 2.8	− 3.4	− 3.2	− 3.3	− 3.3
6	5, 7, 4′-trihydroxyflavone	− 9.8	− 7.8	− 10.3	− 10.3	− 9.2	− 7.5
7	3,3',4',5,5',7-hexahydroxyflavone	− 2.9	− 3.1	− 3.3	− 3	− 3.3	− 3.2
8	Ajoene	− 9.2	− 6.4	− 9.8	− 9.8	− 9.3	− 7.7
9	Allitridin	− 4.2	− 3.1	− 3.3	− 3.7	− 3.4	− 3.2
10	Allyl disulfide	− 3.5	− 2.8	− 2.9	− 4	− 4.3	− 3.3
11	Allyl monosulfide	− 3.1	− 2.8	− 3.1	− 3.3	− 3.2	− 3.1
12	Aloeemodin	− 8.5	− 6.6	− 7.4	− 9.9	− 6.6	− 6.2
13	Anacardic acid	− 3.4	− 2.4	− 3.9	− 7.4	− 3.8	− 4.5
14	Apigenin	− 8.7	− 6.5	− 7.6	− 10.3	− 7.6	− 7.4
15	Archin	− 8.6	− 5.8	− 10.6	− 8.2	− 7.6	− 6.3
16	Aristololactam A IIIa	− 6.7	− 6.4	− 6.9	− 6.9	− 7.4	− 6.7
17	Arundine	− 3.3	− 2.8	− 3.2	− 3.9	− 4.1	− 3.1
18	Atractylenolide i	− 8.3	− 5.8	− 7	− 6.9	− 7.4	− 7.1
19	Atropin	− 7.2	− 5.1	− 6.3	− 6.7	− 7.4	− 6.4
20	Azadirachtin A	− 5.6	− 5.6	− 7	− 7.2	− 7.5	− 7
21	Baicalein	− 8.5	− 8.2	− 10	− 9.8	− 9.7	− 6.8
22	Berberine	− 5.9	− 6	− 6.9	− 6.9	− 6.7	− 7
23	Beta-elemene	− 5.7	− 5.2	− 6.2	− 5.1	− 5.5	− 6.2
24	Betulic acid	− 7.3	− 6.2	− 8	− 7.5	− 7.9	− 6.8
25	Caffeine	− 5.6	− 4.7	− 5.3	− 6.5	− 4.9	− 5.3
26	Calycosin	− 6.9	− 6.4	− 7.5	− 9.9	− 7.8	− 7.4
27	Cambogin	− 6.5	− 7.3	− 7.8	− 7.2	− 7.7	− 8.4
28	Cantharidin	− 5.7	− 5.1	− 6.1	− 7.7	− 7	− 6
29	Capsaicin	− 5.7	− 6.2	− 5.6	− 5.4	− 6.1	− 5.2
30	Casticin	− 7.6	− 7.2	− 7	− 9	− 7.7	− 7.9
31	Catechin	− 7.5	− 6	− 6.8	− 6.9	− 7	− 6.2
32	Celastrol	− 7.4	− 7.1	− 7.8	− 8.2	− 8.7	− 9.5
33	Chelidonine	− 7.9	− 7.5	− 11.1	− 8.3	− 8.3	− 8.2
34	Chrysazin	− 6.7	− 6.7	− 6.8	− 7.3	− 7	− 7.1
35	Citric acid	− 4.3	− 4.8	− 4.7	− 5.4	− 5.7	− 4.2
36	Colchine	− 5.9	− 5.7	− 6.5	− 7.6	− 6.4	− 7.1
37	Coumestrol	− 9.2	− 7	− 10.4	− 10.4	− 7.1	− 7.5
38	Cryptotanshinone	− 7.2	− 6.9	− 7.3	− 7.7	− 7	− 7.7
39	Cucurbitacin b	− 7.5	− 5.8	− 7.1	− 6.8	− 8.1	− 8.7
40	Cucurbitacin e	− 6	− 6.2	− 7.8	− 7.4	− 7.1	− 6.9
41	Curcumine	− 5.5	− 4.8	− 6.7	− 5.8	− 5.4	− 5.2
42	Delta(9)-tetrahydrocannabinol	− 7.5	− 6.2	− 7.1	− 7.2	− 8.9	− 6.8
43	Deoxyelephantopin	− 7.1	− 6.2	− 7.5	− 7.5	− 7.1	− 7.7
44	Deoxyuridine	− 5.9	− 5.4	− 5.8	− 7.5	− 6.9	− 5.9
45	Diallyldisulfide	− 3.5	− 2.7	− 3.2	− 3.3	− 4.3	− 3.3
46	Dihydrotanshinone i	− 9.3	− 7	− 11.2	− 11.2	− 8.2	− 8
47	Dithioerythritol	− 3.1	− 2.7	− 3.6	− 3	− 2.7	− 3
48	Ellipticine	− 9.7	− 6.5	− 7.6	− 8.2	− 7.5	− 7.9
49	(-)-epigallocatechin-3-gallate	− 7.1	− 7.2	− 8.2	− 8	− 8.9	− 9.4
50	Ethyl aldehyde	− 2.5	− 2.2	− 2.5	− 2.3	− 2.5	− 2.2
51	Eupatilin	− 8.8	− 6.1	− 9	− 7.1	− 7.1	− 7.7
52	Ferrum	− 1.2	− 1.1	− 1.3	− 1.3	− 1.3	− 1.1
53	Flavopiridol	− 6.4	− 6.3	− 6.6	− 6.9	− 7	− 7.4
54	Galangin	− 8.4	− 6.1	− 7.3	− 9.6	− 9.5	− 7.6
55	Genistein	− 8.6	− 6.2	− 9.4	− 6.6	− 7.2	− 7.8
56	[4]-gingerol	− 4.2	− 3.4	− 4.6	− 5.4	− 5.5	− 3.7
57	[6]-gingerol	− 3.9	− 4.4	− 4.5	− 4.2	− 4.8	− 4.2
58	Glutathion	− 4.4	− 4.4	− 4.8	− 4.1	− 5.6	− 4.1
59	Glycerin	− 3.5	− 3.6	− 3.6	− 3.5	− 3.5	− 3.7
60	Gossypin	− 6.9	− 6.5	− 7.8	− 9.6	− 6.9	− 8.1
61	Hanfangchin a	− 6.9	− 7	− 8.5	− 8.5	− 9	− 9.5
62	Hesperidin	− 9.2	− 7.5	− 9.2	− 8.4	− 8.6	− 9.5
63	Honokiol	− 5.7	− 5.5	− 9.4	− 6.7	− 8.7	− 6.1
64	Indirubin	− 8.7	− 7.1	− 7.6	− 10.3	− 7.3	− 7.7
65	Isopsoralen	− 7	− 5.6	− 6.4	− 7.9	− 5.9	− 6.3
66	Isorhamnetin	− 6.8	− 6.3	− 7.6	− 7.4	− 7.2	− 7
67	Kaemferol	− 8.6	− 6	− 7.2	− 7.6	− 9.1	− 7.6
68	Kaempferol-7-o-glucoside	− 6.9	− 6.1	− 9.2	− 9.3	− 9.5	− 7.8
69	L20	− 10	− 7.6	− 9.1	− 11.7	− 6.8	− 6.9
70	L-alpha-amino-delta-hydroxyvaleric acid	− 3.6	− 3.2	− 4.4	− 3.8	− 3.4	− 3.8
71	Licochalcone B	− 6.5	− 5.7	− 9.4	− 7.2	− 6.4	− 6.8
72	Lovastatin	− 6.1	− 6.1	− 6.7	− 7.1	− 7.1	− 7.2
73	Lupan-3-one	− 6.8	− 6.7	− 8	− 8.3	− 8.5	− 8.3
74	Lupeol	− 7.5	− 6.3	− 7.2	− 7.9	− 7.5	− 7.7
75	Luteolin	− 6.9	− 6.3	− 10.5	− 10.5	− 9.3	− 7.8
76	Lycopene	− 5.2	− 5.7	− 5.4	− 6.2	− 6.6	− 6.6
77	1,4-naphthoquinone	− 7.2	− 5.2	− 5.7	− 6.5	− 6	− 6.3
78	Nicotine	− 5	− 4.4	− 5.2	− 5.2	− 5.8	− 4.4
79	Oridonin	− 7.2	− 6.7	− 7.4	− 7.5	− 7.3	− 7.9
80	Oroxylin a	− 8.5	− 6	− 9.6	− 6.8	− 6.7	− 7.7
81	Periplocin	− 7.8	− 6.7	− 7.6	− 7.3	− 8.1	− 8.3
82	Progesterone	− 6.2	− 6.6	− 7.2	− 7	− 8.2	− 7.1
83	Protopine	− 7.7	− 6.6	− 7.7	− 7.8	− 7.8	− 8
84	Pseudolaric acid B	− 6.3	− 6.3	− 7.3	− 7.1	− 7.4	− 7.5
85	Puerarin	− 7	− 6.3	− 7.6	− 7.4	− 7.6	− 8.5
86	Quercetin	− 8.2	− 6.6	− 9.9	− 9.3	− 7.8	− 7.4
87	Resveratrol	− 5.7	− 5.5	− 8.9	− 6.4	− 6	− 6.1
88	Secalonic acid d	− 7.7	− 7.4	− 7.5	− 8.3	− 8.3	− 8.9
89	Silybin b	− 7.3	− 7	− 7.5	− 8.3	− 6.9	− 7.5
90	Silydianin	− 6.9	− 6.6	− 7.5	− 7.7	− 7.6	− 7.2
91	Silymarin	− 7.2	− 7.2	− 7.8	− 7.5	− 7.8	− 8.6
92	S-methylmercapto-l-cysteine	− 2.9	− 3.3	− 2.9	− 3.7	− 3.4	− 3.5
93	Sulforathane	− 3.8	− 2.9	− 2.9	− 3.3	− 3.2	− 3.2
94	Tanshinone i	− 10.1	− 7.1	− 8.5	− 11.2	− 7.8	− 8.2
95	Taxol	− 7.3	− 6.3	− 7.7	− 8.9	− 8.3	− 8.9
96	Trans-resveratrol	− 5.3	− 5.4	− 6.1	− 8.3	− 7.2	− 6.5
97	Triptolide	− 6.6	− 6.1	− 7.2	− 7.5	− 7.7	− 7.7
98	Uccinic acid	− 3.8	− 2.8	− 4	− 3.7	− 4.9	− 4.2
99	Ursolic acid	− 7.4	− 6.5	− 7.8	− 7.4	− 7.7	− 9
100	Vanilloid	− 4.4	− 5.3	− 6.2	− 6.3	− 5.4	− 5.9
101	Vinblastine	− 5.4	− 5.6	− 7	− 6.3	− 7.5	− 7.5
102	Withaferin a	− 8.1	− 6.7	− 7.4	− 8.2	− 8.1	− 8.4
103	Wogonin	− 8.6	− 6	− 9.9	− 8.8	− 9.4	− 7.3
104	Xanthotoxin	− 7.3	− 5.5	− 8.3	− 5.7	− 5.7	− 6
105	Zerumbone	− 6.5	− 5.4	− 6.4	− 6.1	− 6.2	− 5.9

### Effects of quercetin, celastrol and cantharidin on cellular viability

**Fig. 7:**
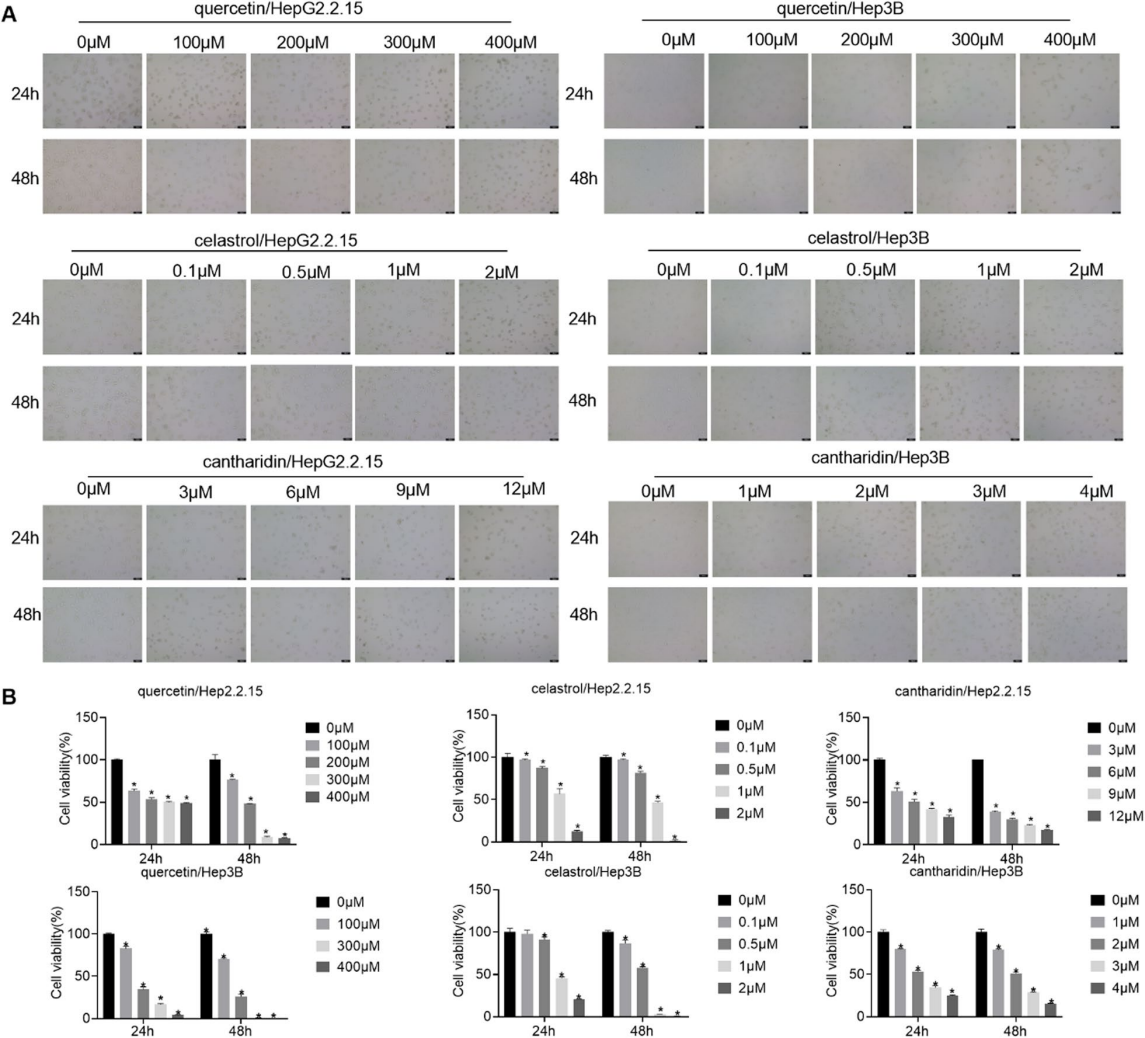
Effects of quercetin, celastrol and cantharidin on cellular viability. A. HepG2.2.15 and Hep3B cells treated with quercetin, celastrol and cantharidin at different concentrations. B. CCK8 was used to detect the cell viability effect of quercetin, celastrol and cantharidin on HepG2.2.15 and Hep3B cells

We further used in vitro experiments to verify the effects of three drugs on two HBV-associated hepatoma cell lines, HepG2.2.15 and Hep3B. HepG2.2.15 and Hep3B cells were treated with quercetin, celastrol and cantharidin for 24 and 48 h, respectively. The results showed that quercetin, celastrol and cantharidin inhibited proliferation of HepG2.2.15 and Hep3B cells, respectively. The IC50 values of quercetin, celastrol and cantharidin in HepG2.2.15 cells were 323.29 μM, 1.24 μM and 5.84 μM at 24 h, respectively (Fig. 7A, B). The IC50 values of quercetin, celastrol and cantharidin in Hep3B cells were 165.81 μM, 0.86 μM and 2.14 μM at 24 h, respectively (Fig. 7A, B). Therefore, these concentrations were selected as the intervention concentrations of quercetin, celastrol and cantharidin in subsequent experiments.

## Quercetin, celastrol and cantharidin decreased the expression of CDK1

**Fig. 8:**
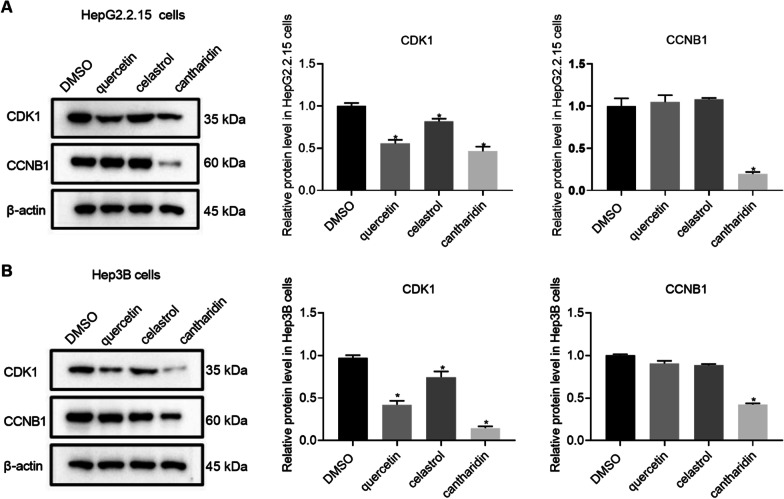
Effects of quercetin, celastrol and cantharidin on the expression of CDK1 and CCNB1. A. Effects of quercetin, celastrol and cantharidin on the expression of CDK1 and CCNB1 in HepG2215 cells. B. Effects of quercetin, celastrol and cantharidin on the expression of CDK1 and CCNB1 in Hep3B cells

Among these top 6 key genes, CDK1 and CCNB1 had the most connection nodes and the highest degree and were therefore, the most significantly expressed. In general, CDK1 and CCNB1 tend to form a complex, which is conducive to cell mitosis [28]. In addition, CDK1 and CCNB1, as cell cycle key proteins, are involved in the development of HBV-related HCC [29]. Hence, we selected two key targets (CDK1 and CCNB1) for further experimental verification. We also selected quercetin, celastrol and cantharidin at the most suitable IC50 concentration for intervention in HepG2.2.15 and Hep3B cells. The results indicated that quercetin, celastrol and cantharidin reduced the expression of CDK1 in HepG2.2.15 and Hep3B cells, respectively (Fig. 8A). Moreover, cantharidin also induced down-regulation of CCNB1 expression in HepG2.2.15 and Hep3B cells, but for CCNB1, only cantharidin decreased CCNB1 expression in the two strains of cells (Fig. 8B).

## Discussion

HBV-related HCC remains one of the major cancers worldwide and has a serious economic impact on healthcare systems. It is important to develop effective diagnosis and treatment strategies and to understand the molecular mechanism of HCC caused by HBV infection.

In this study, three profile datasets were used to identify the DEGs using bioinformatic techniques. We identified a total of 53 upregulated DEGs and 219 downregulated DEGs. Therefore, screening DEGs played an important role in the diagnosis and prognosis of HBV-related HCC. To investigate the interaction of DEGs, PPI network was established and 6 key genes were identified, namely AURKA, BIRC5, CCNB1, CDK1, CDKN3 and TYMS. Moreover, high expression of AURKA, BIRC5, CCNB1, CDK1, CDKN3 and TYMS was associated with poor prognosis in patients with HBV-related HCC. All of the above analyses indicated that CDK1 and CCNB1 might be the most critical targets in HBV-related HCC.

Cyclin‑dependent kinase 1(CDK1), a member of the Ser/Thr protein kinase family, was the most critical cell cycle element for cell proliferation and organ development [30]. G2/mitotic-specific cyclin-B1 (CCNB1) was a major activator of CDK1 [31]. Studies showed that HBV activated CCNB1-CDK1 kinase in HCC cells in vitro [32]. CCNB1 and CDK1 were upregulated in HCC tissues of HBV-positive patients [33], and overexpression of CCNB1 and CDK1 was associated with poor prognosis [34]. In our study, CDK1 and CCNB1 were upregulated and high expression of CDK1 and CCNB1 were associated with poor prognosis in patients with HBV-related HCC. Therefore, we also focused on the verification of CDK1 and CCNB1 in the subsequent experimental verification.

In addition, other targets also play key roles in the development of HBV-related HCC. Aurora kinase A (AURKA) was involved in regulating the G2/M cell cycle and an important predictor of early HCC development [35]. AURKA was up-regulated in HCC tissues and correlated with pathological stage and distant metastasis. Silencing AURKA inhibited radiation-enhanced hepatocarcinoma cell invasion [36]. AURKA lle31Phe enhanced the predisposition of HBV-infected individuals to develop liver cancer [37, 38]. In this study, we found that AURKA was upregulated in many databases. Baculoviral IAP repeat-containing protein 5 (BIRC5) was upregulated in liver cancer. High expression of BIRC5 was associated with poor OS time. Studies showed that OCT4 upregulated the expression of BIRC5 and CCND1 by increasing the promoter activity of BIRC5 and CCND1, promoting HCC cells proliferation [39, 40]. In addition, Thymidylate synthase (TYMS) was an oncogene that regulates the cell cycle. TYMS was associated with HCC metastasis. The silencing of TYMS significantly inhibited the growth and invasion of HCC cells [41]. Our study also found that TYMS was upregulated in HBV-related HCC tissues. Thus, we will continue to study in the future. AURKA, BIRC5, CDKN3 and TYMS may be potential targets for the treatment of HBV-related HCC.

Clinical targeted drugs include sorafenib, palbociclib and Dasatinib. Sorafenib is a multi-target tyrosine kinase inhibitor that promotes apoptosis, reduces angiogenesis and inhibits tumor cell proliferation. Sorafenib is currently an effective first-line therapy in advanced HCC [42]. Studies showed that anti-CDK1 therapy enhanced the anti-tumor response of sorafenib in HCC patient derived xenograft (PDX) model, and provided a reasonable combination therapy to improve the clinical efficacy of sorafenib [43]. Similarly, in our study, we found that sorafenib inhibited BIRC5, CDK1 and CCNB1 expression. Palbociclib is a selective CDK4/6 inhibitor, which was in early stage clinical trials for advanced HCC [44]. A study indicated that palbociclib induced G2/M cell cycle arrest in HCC by down-regulating the phosphorylation of FOXM1 and its downstream target genes AURKA and CCNB1 [45]. Similarly, we found that palbociclib inhibited AURK1 BIRC5, CCNB1, CDK1, CDKN3 and TYMS expression. Dasatinib is a multi-target protein tyrosine kinase inhibitor that targets the Src kinase family and has been investigated as a targeted therapy for broad-spectrum cancer types. We found that Dasatinib inhibited the expression of AURKA and BIRC5 in HBV-related HCC. Similarly, A study showed that Dasatinib inhibited the proliferation of liver cancer cells [46].

Chemotherapy drugs, such as DNA-targeting cisplatin and doxorubicin, are widely used to treat a variety of cancers, including HCC [9]. Cisplatin cross-links and damages DNA, activates DNA damage response, and subsequently induces apoptosis of cancer cells [9, 47]. In our study, we found that cisplatin increased or decreased these 6 key targets. Similarly, studies indicated that BIRC5 played a key role in promoting cell proliferation and enhancing the sensitivity of liver cancer to chemotherapy. Therefore, inhibition of BIRCR expression in combination with cisplatin might contribute to the development of more effective therapies for liver cancer [48]. Moreover, a study indicated that CDKN3 expression was negatively correlated with tumor staging, and CDKN3 inhibition promoted hepatocellular cancer cell survival and cisplatin tolerance [49]. Doxorubicin is an anthracycline antitumor drug with advantages and a broad spectrum of efficacy [50]. When taken up by cells, Doxorubicin acts primarily through non-covalent inclusion of double-stranded DNA, thereby inhibiting DNA replication in rapidly proliferating tumors [51]. We found that Doxorubicin had an effect on 6 key targets. Similarly, studies showed that CDKN3 silencing did not significantly inhibit the proliferation of HCC, but reduced their sensitivity to Doxorubicin [52]. Furthermore, miR-26a-5p inhibited HCC cells proliferation and enhanced Doxorubicin sensitivity by inhibiting AURKA [53].

In this study, we conducted reverse enrichment of TCM according to the key targets, and analyzed the properties and effects of the drugs. The properties and functions of TCM were the important basis for its clinical application. According to the theories of meridian tropisms, zang-fu, yin and yang, and TCM therapeutic principles, TCM theory was usually summarized as four properties and five tastes, meridian tropism, etc. [25]. Our study found that the properties of TCM treatment for HBV-related liver cancer were mainly warm, followed by bitter cold. Cold herbs were associated with inflammation/immunity modulation, and warm herbs affected cell growth and proliferation, reflecting that TCM herbs were widely involved in immune and cellular processes [54, 55]. Under the framework of TCM theory, bitter cold herbs cleared away heat and removed pathogenic qi. The extracts of *Scutellaria barbata* and Oldenlandia diffusa (Willd.) Roxb inhibited the growth of hepatitis B virus-associated hepatocellular carcinoma by regulating circRNA expression [56]. *Scutellaria barbata* inhibited the expression of key genes and blocked the PI3K-AKT signaling pathway to inhibit the proliferation, and migration and induce apoptosis of liver cancer cells [57]. The warm and sweet herbs had a tonic effect, suggesting that removing deficiency and filling deficiency was the main principle of TCM in the treatment of liver cancer [58]. In addition, in our previous study, we found that the application of Wenshen prescription (composed of Yougui pill and Haoqin Qingdan decoction) achieved good efficacy in the treatment of hepatitis B-related advanced liver cancer patients. The objective tumor response rate and disease control rate in the treatment group were significantly better than those in the control group. The median survival time was 6.74 months in the treatment group and 4.85 months in the control group. The survival time of the treatment group was better than that of the control group [59]. Meridians orientation contributed to understand the direction of action of drugs. In this study, the TCM was usually liver meridian, which was more consistent with the treatment of HBV-related liver cancer, followed by lung, spleen, stomach, further indicating that the role of lung, spleen, stomach and other organs was also equally important.

Then, in this study, several small molecules of TCM were obtained. Flavonoid compounds include quercetin, hesperidin, silymarin, casticin, and so on. Some studies demonstrated that quercetin inhibited the proliferation of liver cancer cells via induction of apoptosis and cell cycle arrest. The representative component quercetin was selected for experimental verification, and it was found that quercetin inhibited the proliferation of HepG2.2.15 cells and Hep3B cells, and reduced the expression levels of CDK1. Similarly, studies showed that quercetin reduced the expressions of CDK1 [60], and inhibited the proliferation of liver cancer [61]. Terpenoids and glycosides include celastrol and ursolic acid. Celastrol inhibited the growth and induced apoptosis of human HCC by regulating the STAT3/JAK2 signaling cascade [62]. It also induced caspase-dependent apoptosis of HCC cells by inhibiting the mammalian target of rapamycin (mTOR) [63]. It was also found in experimental verification that celastrol could inhibit the proliferation of HepG2.2.15 cells and Hep3B cells at a lower concentration. Moreover, we also found that celastrol reduced CDK1 expression.

Alkaloids include cantharidin and berberine. Cantharidin inhibited the proliferation of HCC stem cells and induced cell apoptosis through G2/M phase cell cycle arrest [64]. In this study, we also found that cantharidin inhibited the proliferation of HepG2.2.15 cells and Hep3B cells, and decreased the expression levels of CDK1 and CCNB1. In this study, we focused on the study of three different types of small molecular of TCM on HBV-related HCC, and the research content in this area is relatively small. At the same time, it gives us inspiration to study the effect of newly discovered small molecular of TCM on HBV-related HCC according to the classification of small molecules. In addition, we only selected three representative small molecules for verification. All three small molecules were purchased standard drugs, not self-synthesized drugs, which affected the innovation of the research. In the future, we will continue to discover new anti-HBV-related HCC drugs and conduct chemical synthesis research.

## Conclusion

In summary, this study used key targets to study the TCM treatment of HBV-related liver cancer in a reverse network pharmacology system. We found that clinical drugs including chemotherapy drugs, targeted drugs, small molecules of TCM including flavonoids, terpenoids and glycosides and alkaloids, bitter and warm as well as TCM belonging to the liver and lung meridian might be useful for the treatment of HBV-related HCC. These conclusions provided a new idea for HBV-related HCC.

## Author Statement

We declared that this manuscript is original, has not been published before and is not currently being considered for publication elsewhere.

We confirm that all data were generated in-house, and no paper mill was used. All authors agree to be accountable for all aspects of work ensuring integrity and accuracy.

## Data Availability

The datasets used and/or analyzed during the current study are available from the corresponding author on reasonable request.

## Abbreviations

HCC: Hepatocellular carcinoma
HBV: Hepatitis B virus
HBV-related HCC: Hepatitis B virus-associated hepatocellular carcinoma
TCM: Traditional Chinese medicine
GEO: Gene expression synthesis
GEPIA: Gene expression profile Interaction Analysis
DEGs: Differentially expressed genes
STRING: Search Tool for the Retrieval of Interacting Genes/Proteins
PPI: Protein–protein interaction
TCGA: The cancer genome atlas
GTEx: Genotype-tissue expression
OS: Overall survival
HR: Hazard ratios
LIHC: Liver hepatocellular carcinoma
BLCA: Bladder urothelial carcinoma
BRCA: Breast invasive carcinoma
CHOL: Cholangiocarcinoma
COAD: Colon adenocarcinoma
ESCA: Esophageal carcinoma
HNSC: Head and neck squamous cell carcinoma
KIRC: Kidney renal clear cell carcinoma
LUAD: Lung adenocarcinoma
LUSC: Lung squamous cell carcinoma
STAD: Stomach adenocarcinoma
UCEC: Uterine corpus endometrial carcinoma
BIRC5: Baculoviral IAP repeat-containing protein 5
CDK1: Cyclin‑dependent kinase 1
CCNB1: G2/mitotic-specific cyclin-B1
AURKA: Aurora kinase A
TYMS: Thymidylate synthase
CDKN3: Cyclin-dependent kinase inhibitor 3
CCK-8: Cell Counting Kit-8
